# Low vision rehabilitation in improving the quality of life for patients with impaired vision: A systematic review and meta-analysis of 52 randomized clinical trials: Retraction

**DOI:** 10.1097/MD.0000000000026669

**Published:** 2021-07-16

The article “Low vision rehabilitation in improving the quality of life for patients with impaired vision: A systematic review and meta-analysis of 52 randomized clinical trials”,^[1]^ which published in Volume 100, Issue 19 is being retracted due to academic misconduct. After an investigation into reader concerns, the Medicine Editorial Office found that passages had been directly plagiarized and/or slightly modified from “Low vision rehabilitation for better quality of life in visually impaired adults”.^[2]^ The categorization of interventions, pooling of data, a flow chart, references, and data in forest plots were also plagiarized.

In addition to plagiarism concerns, included studies Stroupe 2018, Stelmack 2017, Brody 2006, and Brody 2002 were misrepresented as four separate studies. Stroupe 2018 belongs to the same study as Stelmack 2017, and Brody 2006 is a subsample of Brody 2002. It was also discovered that authors submitted the publication to *International Journal of Surgery* while it was still under consideration by Medicine. *International Journal of Surgery* withdrew the epublication for similar concerns.^[3]^
